# Analysis of hydration and subchondral bone density on the viscoelastic properties of bovine articular cartilage

**DOI:** 10.1186/s12891-022-05169-0

**Published:** 2022-03-08

**Authors:** Joseph P. Crolla, Bernard M. Lawless, Anna A. Cederlund, Richard M. Aspden, Daniel M. Espino

**Affiliations:** 1 Deptment of Mechanical Engineering, University of Birmingham, Birmingham, B15 2TT UK; 2 Centre for Arthritis and Musculoskeletal Health, University of Aberdeen, AB25 2ZD Foresterhill Aberdeen, UK

**Keywords:** Articular cartilage, Bone density, Hydration, Loss, Storage, Viscoelasticity

## Abstract

**Background:**

Articular cartilage is known to be a viscoelastic material, however little research has explored the impact of cartilage water content and bone density on its viscoelasticity. This study aimed to isolate subchondral bone density and hydration of articular cartilage and analyse their effects on the viscoelastic properties of articular cartilage.

**Methods:**

Dynamic mechanical analysis was used to test samples at frequencies of 1, 8, 12, 29, 49, 71, and 88 Hz. Synthetic bone material with densities of 663.7 kg/m^3^ and 156.8 kg/m^3^ were used to mimic the bone mineral density (BMD). Dehydration occurred in a stepwise manner at relative humidity (RH) levels of 100%, 30%, and 1%. These relative humidity levels led to water contents of approximately 76%, 8.5%, and ≈ 0% by mass, respectively.

**Results:**

Samples from eight bovine femoral heads were tested under a sinusoidal load. Storage stiffness was lower on the lower substrate density. Storage stiffness, though, increased as cartilage samples were dehydrated from a water content of 76% to 8.5%; decreasing again as the water content was further reduced. Loss stiffness was lower on a lower density substrate and decreased as the water content decreased.

**Conclusions:**

In conclusions, a decrease in hydration decreases the loss stiffness, but a non-linear relationship between hydration and storage stiffness may exist. Additionally, higher BMD values led to greater storage and loss stiffnesses.

**Supplementary Information:**

The online version contains supplementary material available at 10.1186/s12891-022-05169-0.

## Background

Articular cartilage is a specialised connective tissue located on the articular surface of bones. Its primary purposes are to create a smooth [1], lubricated surface for low-friction articulation; as well as helping with the transmission of loads to the underlying subchondral bone [2]. Osteoarthritis (OA) is a degenerative disease of the whole joint for which there is currently no cure. However, procedures such as total hip or knee arthroplasty are common in patients with severe OA, with over 200,000 operations being performed in England and Wales and a further 15,000 in Scotland, during 2018[3, 4]. These figures are rising annually, and most procedures are for osteoarthritis. In the USA, about 1.5 million hip and knee joint replacements were predicted for 2020 [5]; with 700,000 being performed in 2012 [6].

Cartilage is made up of approximately 70% water by weight [7]; the water content of cartilage has been reported by Venn & Maroudas to be lower in the deep zone (67%) as compared to the superficial zone (74%) [2]. The amount of water found within articular cartilage decreases with age [8, 9], but there is some evidence that it increases before the onset of osteoarthritis [10]. Removing water from cartilage has been suggested as leading to an increase in strength by aiding in the redistribution of stress from the loading site [11, 12]; whereas an increase in water content is thought to reduce the induced stress required to cause failure when tested under creep conditions [13, 14].

Cartilage exhibits frequency-dependent viscoelastic behaviour [15, 16]. Viscoelasticity can be characterised through a storage and loss modulus [17–19], or via storage and loss stiffness for a structure [20, 21]. Although several studies have assessed the role that frequency [17, 22] and thickness [21] have on the viscoelastic response of articular cartilage, fewer have evaluated the effect of hydration. One such study by Pearson & Espino [20] compared the difference in viscoelastic response between hypo- and hyper-hydrated cartilage-on-bone. That study concluded that the storage stiffness increased with dehydration, with an altered frequency-dependency, whereas the loss stiffness was offset but remained frequency independent with changes in hydration. Those findings were consistent with results from impact and stress relaxation studies performed at two levels of hydration [7], which found an increase in stiffness with reduced water content. Although Pearson and Espino [20] succeeded in varying the water content, their study was qualitative. It is not clear whether the hydration conditions they achieved were physiological or patho-physiological; further, changes in mass water content were not quantified during altered stages of hydration. It is also unclear whether the attachment to the underlying subchondral bone hindered the hydration/dehydration process.

Subchondral bone refers to the layer of bone which directly underlies the articular cartilage in a joint. The most common symptom associated with OA is subchondral sclerosis, which is defined as increased bone density or thickening in the subchondral layer of a joint [23]. This is often the first radiographic sign of OA [24]. Long term changes in the structure of subchondral bone play an important role in osteoarthritis [25–28]. These include changes in bone turnover, mineralisation, and bone volume; resulting in a reduction in bone density, and an overall weaker, less mineralised bone [24, 29]. An increase in density of the subchondral bone in radiographs is used as a clinical sign of radiographic OA [24].

A longitudinal magnetic-resonance imaging study noted that an increase in tibial subchondral bone area preceded an increase in cartilage volume and subsequent cartilage defects [30]. This susceptibility to damage implies interaction between changes to the underlying bone, and physical changes, for example an increase in swelling, in cartilage. Although studies have been conducted on the effect of bone density on cartilage damage [31–33] and recent studies have assessed its effect on the viscoelastic properties of cartilage [34, 35] and failure due to loading frequency, no studies have assessed whether changes to hydration and bone density may alter the storage and loss stiffnesses of cartilage.

The aim of this study is to quantitatively assess the effect of hydration and substrate density, as independent variables, on the viscoelastic properties of articular cartilage. Dynamic Mechanical Analysis (DMA) has been used to characterise storage and loss stiffness following controlled variation of the water content of bovine cartilage, and of the density of the underlying substrate. Two experimental procedures were used to test these variables independently. Water content was varied using a humidity chamber which achieved a variation in humidity of 1 to 100%, which allowed for a difference in cartilage water content of 0 – 76%. These values were chosen to allow testing to be performed that would isolate the effect of water on the viscoelastic properties. A value of 8.5% was included in between to show the effect of dehydration under less extreme conditions than 0% water content. Substrate density was varied by using two bone mimicking materials manufactured by Sawbones (Malmö, Sweden), with densities of 663.7 kg/m3 and 156.8 kg/m^3^. These two materials have been commercially manufactured as models for cortical and trabecular bone.

## Materials and methods

### Sample preparation

Cartilage samples (*n* = 56) from eight bovine femoral heads, approximately between 18 and 30 months old were obtained from a supplier (Dissect Supplies, Birmingham, UK). Seven samples were harvested at random from the equator of each femoral head, avoiding the insertion of the ligament, and the apex of the femoral head in order to remove regional differences as a variable; regional variations in dynamic moduli are known to occur across joints [34]. India ink was used to highlight and avoid damaged areas of the articular surface during sample preparation. For hydration testing, 5.2 mm diameter, full-depth cartilage samples were obtained manually using a cork borer, consistent with previous studies [21, 22]. For tests assessing bone density, cartilage samples were obtained using a 6 mm cork borer, this matched the diameter of all artificial Sawbone samples. These Sawbone samples were obtained using a pillar drill with a 6 mm diameter core drill bit. The effect of hydration and substrate density were tested independently, with 24 samples used for substrate testing (3 from each joint) and 16 samples used for hydration testing (2 from each joint). Eight test specimens (1 from each joint) were used as control samples for substrate density test procedures; a further 8 independent samples were used as control samples for the hydration study.

### Dynamic mechanical analysis

DMA was performed by applying a sinusoidal load. The resulting sinusoidal displacement was measured, and the phase angle between the two determined. Fast Fourier Transforms (FFT) of both the load (F) and displacement (d) were used to calculate the dynamic stiffness (k*) as a ratio of the magnitudes of the two FFT data-length sets, i.e. F* and d* respectively (Eq. 1); further explained elsewhere[18]. The storage (${\mathrm{k}}^{\mathrm{^{\prime}}})$ and loss $({\mathrm{k}}^{\mathrm{^{\prime}}\mathrm{^{\prime}}}$) stiffness are then calculated according to Eqs. 2 and 3, noting that *δ* represents the phase lag between the applied load and the resulting displacement.

1 $${k}^{*}= \frac{{F}^{*}}{{d}^{*}}$$

2 $${k}^{\mathrm{^{\prime}}}={k}^{*}\mathrm{cos}\left(\delta \right)$$

3 $${k}^{\mathrm{^{\prime}}\mathrm{^{\prime}}}={k}^{*}\mathrm{sin}\left(\delta \right)$$

### Frequency sweep

A materials testing machine (Bose Corporation, ElectroForce Systems Group, Minnesota, USA) was used to perform DMA over a frequency sweep of 1, 8, 10, 12, 29, 49, 71, and 88 Hz [17, 18, 20, 21, 34]. Two preconditioning frequencies of 25 and 50 Hz with a 60 s rest period were also used [17]. The load applied induced a nominal compressive stress which varied sinusoidally from 0.75 – 1.7 MPa; where 1.7 MPa is anticipated as a peak physiological stress during ‘ambulatory’ activity [36] (i.e. walking). The applied load was adjusted according to the two main specimen *en face* surface areas, to ensure they were tested at the same nominal level of induced stress.

### Substrate protocol

**Fig. 1:**
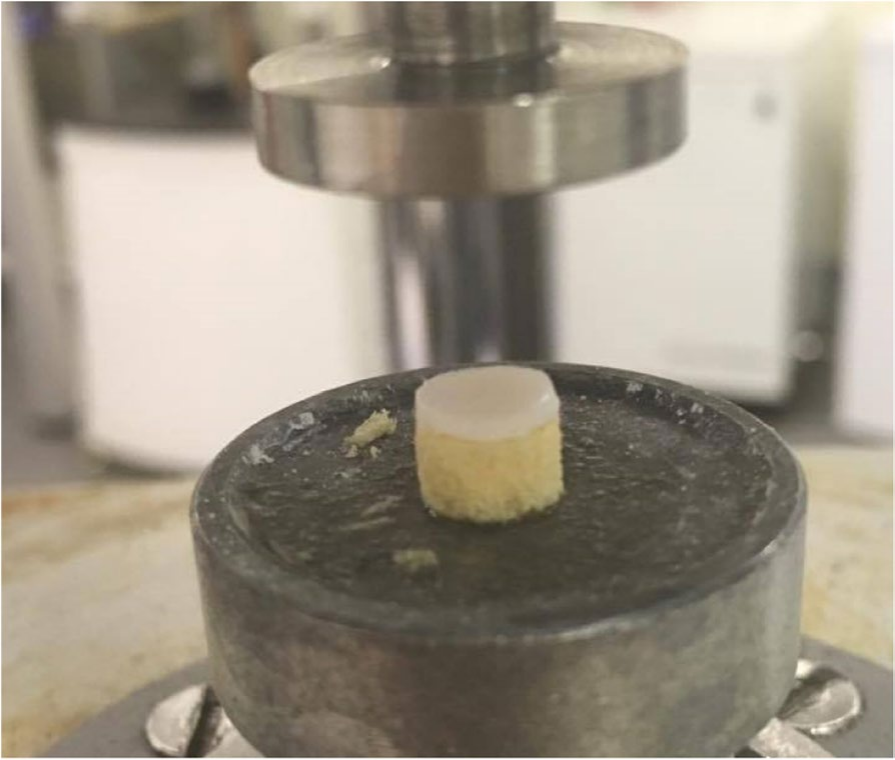
Cartilage and substrate (Sawbone core) set-up on testing machine (compression plate)

A simplified cartilage-on-bone model was used, in which the cartilage was not bound to the synthetic material. Instead, cartilage samples were simply placed on top of the bone substitute [35] (Fig. 1). Each of the 24 samples was tested twice, on both a high- and a low-density synthetic bone material.

To reduce bias, half the samples were tested on the high-density material first, whilst the other half were tested first on the lower density material. Two rigid polyurethane foams (Sawbones Europe AB, Malmoe, Sweden) of densities 663.7 kg/m^3^ and 156.8 kg/m^3^ and thickness of 4 mm were used. All specimens used to test the effect of substrate density were stored in Ringer’s solution, to ensure a constant hydration level; there was a gap of 4 h between tests. In addition, eight control samples were tested twice with no substrate, to quantify the effect of repeat testing on a single sample.

### Hydration protocol

**Fig. 2:**
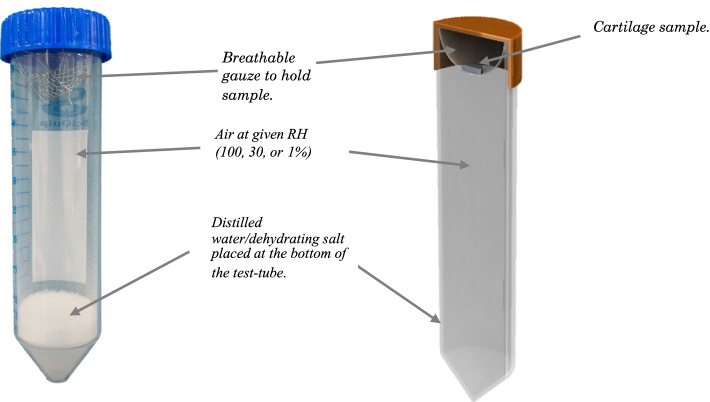
Image (**a**) and cross-sectional diagram (**b**) of hydration chamber

All twenty-four specimens were stored in distilled water following dissection (Sect. 2.1). This ensured that the cartilage samples would be fully hydrated before a dehydration procedure using a hydration chamber. For the initial phase of testing at a Relative Humidity of 100% (RH-100%), 40 ml of distilled water was placed in a test tube, and a cartilage sample was suspended above using a breathable plastic gauze **(**Fig. 2).

This allowed free movement of air between the distilled water and cartilage sample, allowing an equilibrium RH-100% to be achieved. Hydration to experimental stages of relative humidity were achieved by placing samples in hydration chambers with 20 g of Sodium Chloride (NaCl), or 5 g of Magnesium Chloride (Sigma Aldrich, St Louis, Missouri, USA); achieving RH-30% and RH-1% respectively. These conditions mimicked maximal hydration, dehydration and full dehydration of the cartilage sample. A control apparatus was set up using a capacitive hygrometer (Thermo Fischer Scientific, Waltham, Massachusetts, USA). This allowed monitoring of the relative humidity throughout the dehydration process. The hygrometer used had an accuracy of 1.5%, and a resolution of 0.01%. Eight control samples were maintained at RH-100% throughout testing. All samples were weighed after each stage using an Ohaus GA200D digital scale (OHAUS Corporation, Parsippany, New Jersey, US) and the water loss calculated from the changes in mass.

Sixteen samples from eight bovine femoral heads were tested and then dehydrated in a stepwise manner over the course of three days. The samples were initially hyper-hydrated at RH-100% for 24 h before initial DMA. They were then dehydrated and tested two more times at RH-30% and RH-1% in 24-h time steps. After the last DMA experiment was completed, samples were replaced in hydration chambers at RH-1% to allow them to fully dehydrate and calculate water content at each step. Eight control samples were stored in hydration chambers at RH-100% for the entire three-day period and tested three times in 24-h increments.

### Data analysis

Data are presented as mean ± standard deviation. Confidence intervals were calculated using the number of independent observations[37] (*N* = 8). As this study used repeated measurements on a single sample, a paired non-parametric statistical test (Wilcoxon Signed Rank) was used to explore changes in stiffness caused by substrate density. One-way ANOVA was used to assess differences in stiffness across the three hydration levels at all frequencies. The trendlines of regression fitted had the form shown in Eqs. 5 and 6, for ${\mathrm{k}}^{\mathrm{^{\prime}}}$ and ${\mathrm{k}}^{\mathrm{^{\prime}}}\mathrm{^{\prime}}$ respectively, consistent with previous studies [20, 34]. SigmaPlot 13.0 (Systat Software Inc., Chicago, Illinois) was used for all statistical analysis.

5 $${k}^{\mathrm{^{\prime}}}=A ln\left(f\right)+B$$

6 $${k}^{\mathrm{^{\prime}}\mathrm{^{\prime}}}=C ln\left(f\right)+D$$

Here *A* and *C* denote the gradient of the storage and loss stiffness with respect to the natural logarithm of the frequency *(f)*; *B* and *D* denote an intercept.

Data obtained from the testing of control samples are provided in a Supplementary data file. This includes results for repeat testing under substrate (Section S.1) and hydration (Section S.2). To avoid repetition in the results section, but to ensure data are accessible, tabulated data sets obtained from testing are provided as Supplementary data (Section S.3).

## Results

### Substrate density

**Fig. 3:**
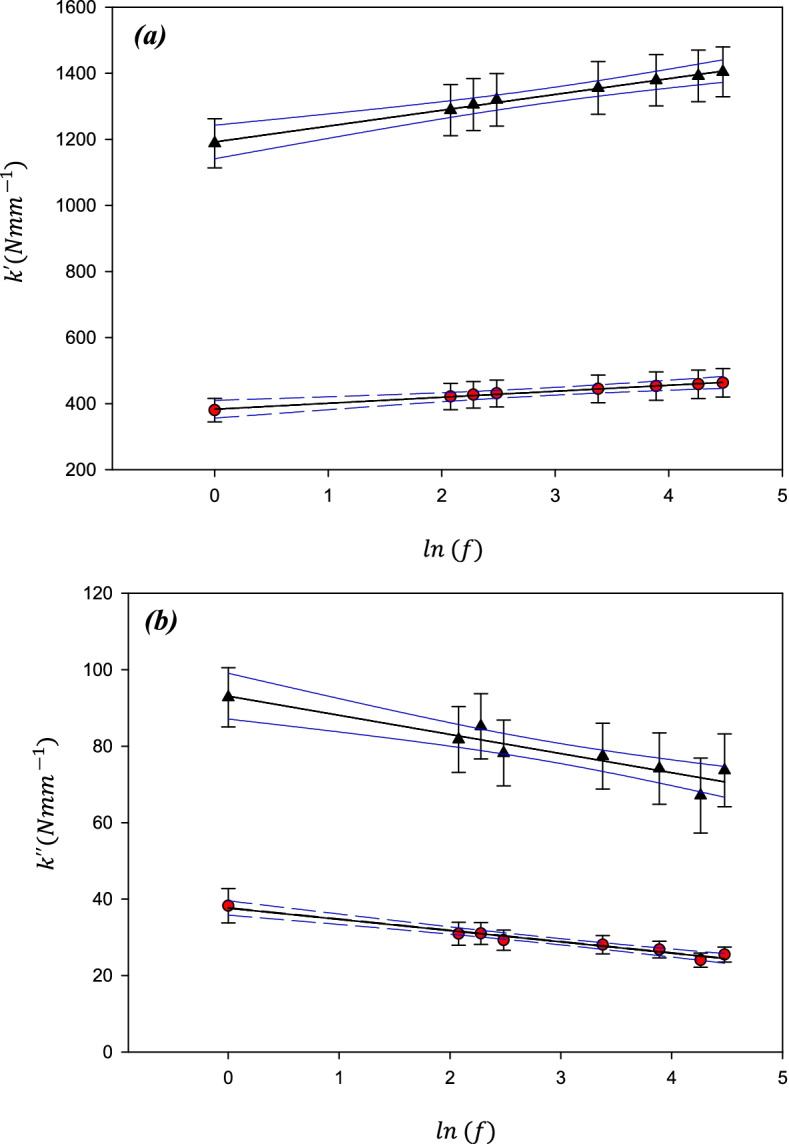
Mean stiffness as a function of the Natural log(frequency; f) for (**a**) Storage stiffness and (**b**) loss stiffness of cartilage-on-Sawbone samples on substrates with two different densities; 663.7 kg/m3 (black triangles) and 156.8 kg/m3 (red circles). Mean values have been calculated from *n* = 24 samples. Error bars show 95% confidence intervals for *N* = 8 independent samples, blue lines show 95% confidence intervals for the lines of regression for *N* = 8 independent samples

At all frequencies, the storage stiffness, ${\mathrm{k}}^{\mathrm{^{\prime}}}$, was lower on the lower density substrate than on the high-density substrate. On the low-density material, it ranged from 380 ± 45 N/mm at 1 Hz, to 463 ± 54 N/mm at 88 Hz (hereon reported in the form: 380–463 N/mm at 1–88 Hz). On a higher density substrate the mean value of ${\mathrm{k}}^{\mathrm{^{\prime}}}$ increased (1182–1397 N/mm at 1–88 Hz). A linear relationship was found between ${\mathrm{k}}^{\mathrm{^{\prime}}}$ and the natural logarithm of the frequency (Fig. 3a). The frequency dependency of ${\mathrm{k}}^{\mathrm{^{\prime}}}$ i.e. *A*, the gradient of the regression line (*Eq. **5*), was lower for the lower density (18.1 ± 2.3 N/mm) compared with that on the higher density (47.8 ± 4.8 N/mm) substrate.

Similarly, ${\mathrm{k}}^{\mathrm{^{\prime}}\mathrm{^{\prime}}}$ was consistently lower at each frequency on the lower density substrate than on the higher density substrate but decreased as the frequency increased (Fig. 3b). Mean values of ${\mathrm{k}}^{\mathrm{^{\prime}}\mathrm{^{\prime}}}$ for the cartilage tested on a low-density substrate (38–25 N/mm at 1–88 Hz) were lower than for cartilage tested using a high-density substrate (93–74 N/mm at 1–88 Hz)**.**

### Hydration

**Fig. 4:**
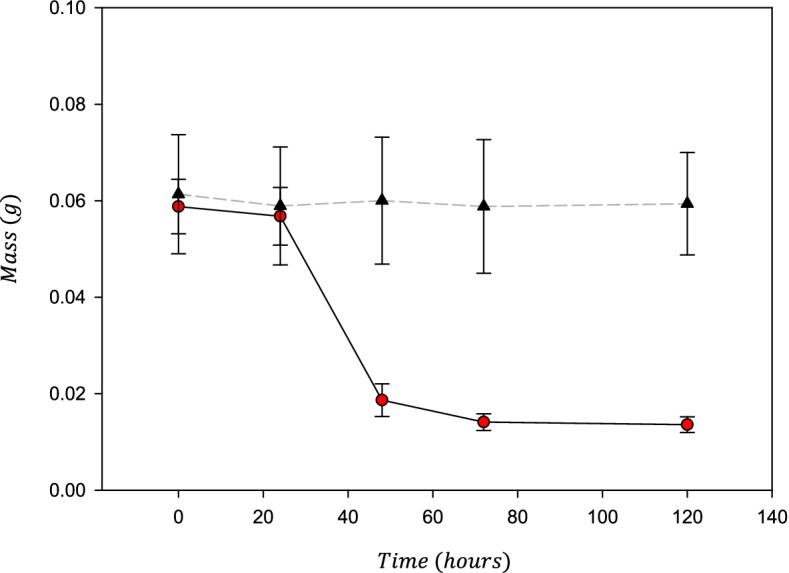
Mass of cartilage samples measured after 24, 48, 72, and 120 h. Control samples (black triangles, dashed line) were kept in an RH of 100%, whilst other samples were dehydrated in a step-wise manner from 100 to 30% to 1% every 24 h (red circles, solid line) before being kept in RH 1% for a further 48 h to fully dehydrate. Error bars show 95% confidence interval for *N* = 8 independant samples

The mass of the cartilage decreased as RH was reduced from 100 to 30%; with an average loss of 67.8 ± 4.9% mass. Samples lost an additional 7.4 ± 4.7% of their original mass when subsequently dehydrated to RH-1%. Dehydration for an additional 48 h led to a further reduction of 0.9 ± 1.0% of the original mass (Fig. 4) Accordingly, the mean total water content was 76% by mass, reducing to 8.5% at RH-30% and effectively 0% at RH-1%.

**Fig. 5:**
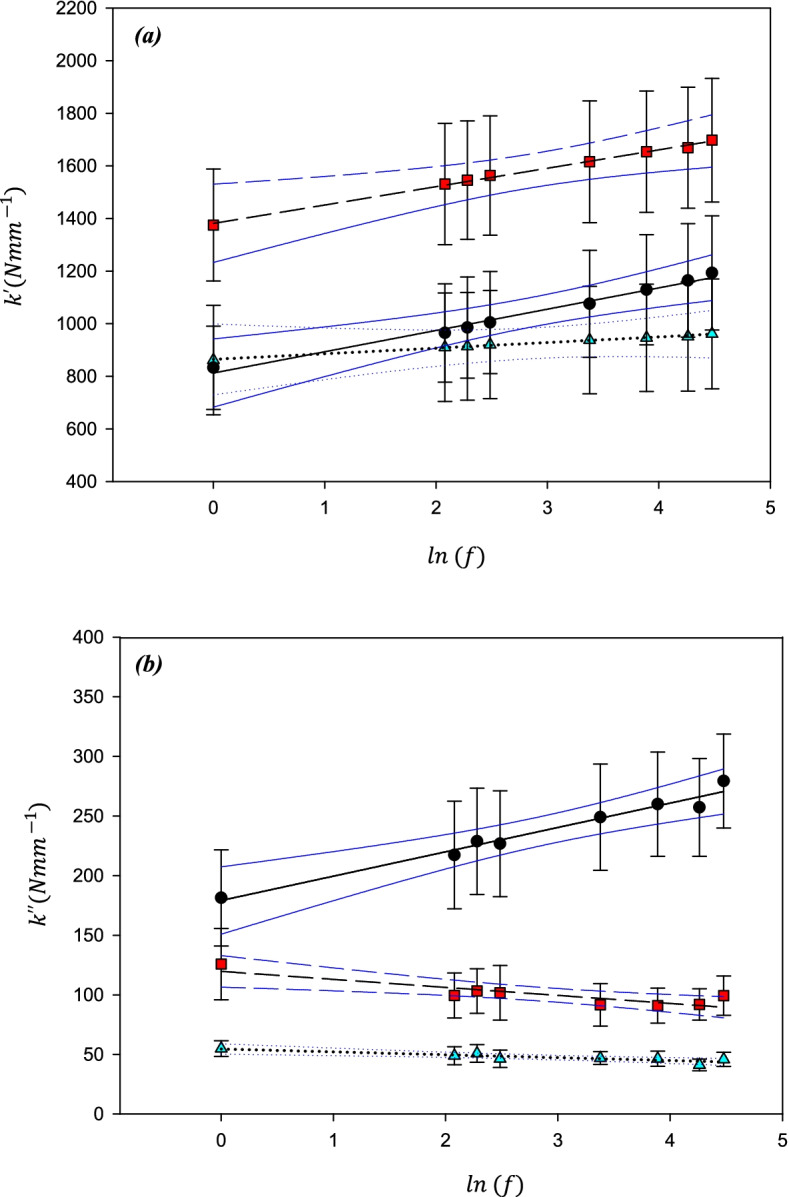
Mean stiffness as a function of the Natural log(frequency) for **(a)** Storage stiffness and **(b)** loss stiffness of cartilage samples at three different hydration levels; RH-100% (black circles), RH-30% (red squares), and RH-1% (cyan triangles). Mean values have been calculated from *n* = 16 samples. Error bars show 95% confidence intervals for *N* = 8 independent samples, blue lines show 95% condidence intervals for the lines of regression for *N* = 8 independent samples

**Fig. 6:**
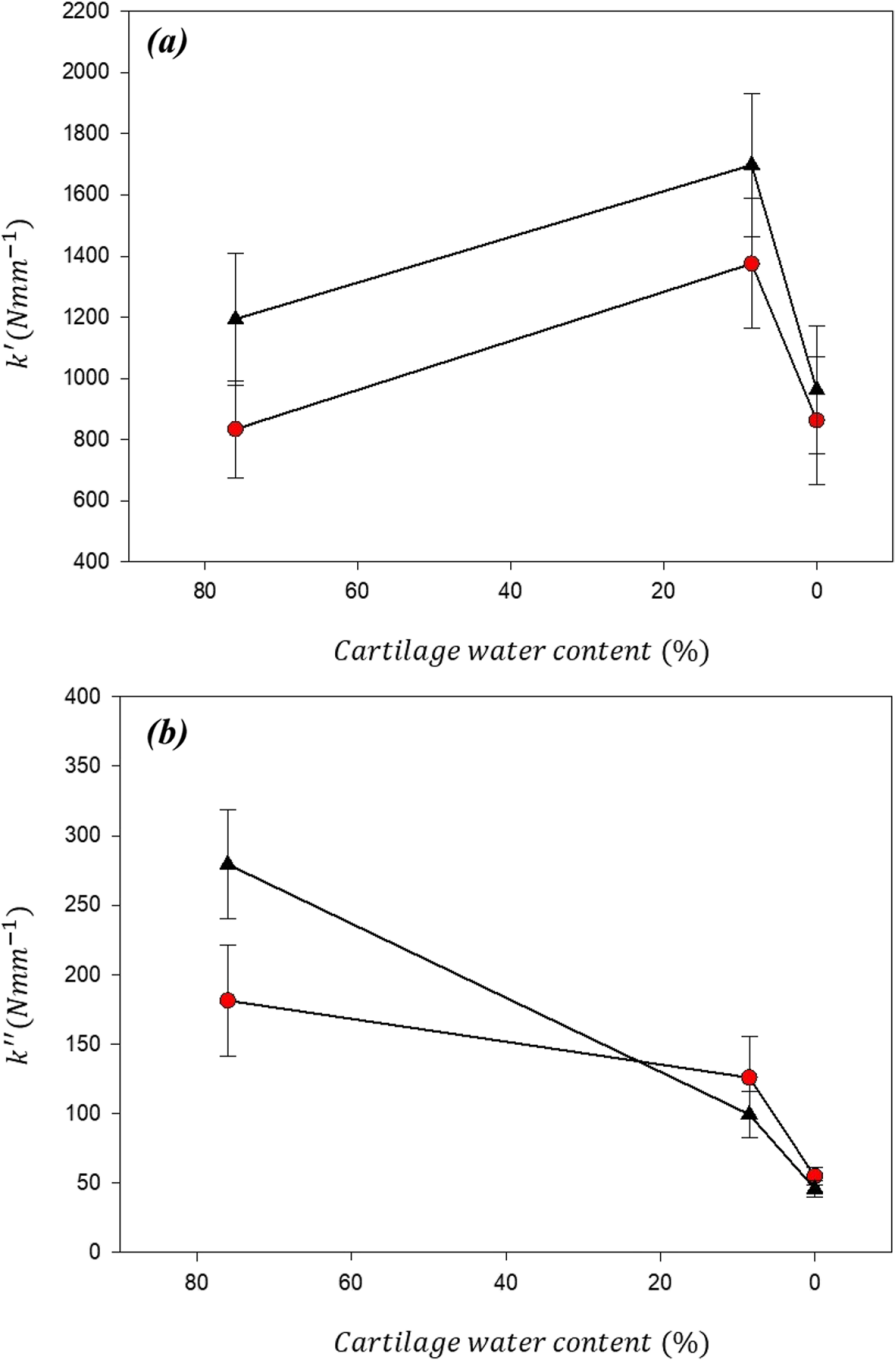
The variation of storage (**a**) and loss (**d**) moduli with respect to cartilage water content at 1 Hz (red circles) and 90 Hz (black triangles). Error bars show 95% confidence intervals

The storage stiffness, ${\mathrm{k}}^{\mathrm{^{\prime}}}$, of articular cartilage was significantly greater at all frequencies when (de)hydrated at RH-30% (1375–1698 N/mm at 1–88 Hz) as compared to RH-100% (832–1193 N/mm at 1–88 Hz). Further reduction in water content between RH-30% and RH-1%; resulted in a reduction in storage stiffness at all frequencies but also some loss of frequency dependency (RH-1%: 862–961 N/mm at 1–88 Hz). There was no significant difference in ${\mathrm{k}}^{\mathrm{^{\prime}}}$ between RH-100% and RH-1% at any of the frequencies used for testing (Figs. 5a and 6a). *A* decreased with RH, being 14% lower at RH-30% compared with RH-100%, and 74% lower at RH-1%.

In contrast to the storage stiffness, dehydration not only resulted in a reduction in k″ at all frequencies but also a change in the sign of the gradient of the frequency dependency, at lower values of hydration. The range for ${\mathrm{k}}^{\mathrm{^{\prime}}\mathrm{^{\prime}}}$ for RH-100% (181–279 N/mm at1-88 Hz) was greater than at RH 30% (126–99 N/mm at 1–88 Hz) and was lowest for RH-1% (55–46 N/mm at 1–88 Hz). The change in frequency-dependency with hydration (Figs. 5b and 6b) is quantifiable by a reduction in *C* (*Eq. **6*) from 20.4 N/mm at RH-100%, as compared to -6.7 N/mm for RH-30% and -2.4 N/mm at RH-1%.

## Discussion

This study shows that water content and substrate density affect both the storage and loss stiffnesses of articular cartilage. The results indicate that the storage stiffness, ${\mathrm{k}}^{\mathrm{^{\prime}}}$, increases with reducing tissue hydration (from 76% to 8.5% water mass fraction); but subsequently reduces as the tissue water content continues to reduce. Because, in this study, only three levels of hydration were used, we are unable to say at what level of hydration a turning point occurs. The stage of RH-1% represented a quasi-dehydrated state (approximating ≈ 0% water content). These findings suggest a non-linear relationship between ${\mathrm{k}}^{\mathrm{^{\prime}}}$ and hydration for articular cartilage. A different trend was apparent when comparing ${\mathrm{k}}^{\mathrm{^{\prime}}\mathrm{^{\prime}}}$ and water content of cartilage; with a reduction in ${\mathrm{k}}^{\mathrm{^{\prime}}\mathrm{^{\prime}}}$ seen for all samples when the water content was decreased. Increasing the substrate density resulted in an increase in both ${\mathrm{k}}^{\mathrm{^{\prime}}}$ and ${\mathrm{k}}^{\mathrm{^{\prime}}\mathrm{^{\prime}}}$ of the cartilage sample. Thus, this study suggests that an increase in bone density increases osteochondral dynamic stiffness. If it is important for the osteochondral construct to maintain a constant stiffness. A response to counteract any initial increase in substrate stiffness (caused by sclerosis of bone) could be through increased swelling as this study has shown that a decrease in water content may result in an increase of ${\mathrm{k}}^{\mathrm{^{\prime}}}$.

This study has shown that there may be a non-linear relationship between hydration and ${\mathrm{k}}^{\mathrm{^{\prime}}}$. ${\mathrm{k}}^{\mathrm{^{\prime}}}$ increased as tissue water content decreased 76% to 8.5%. For both 76% and 8.5%, ${\mathrm{k}}^{\mathrm{^{\prime}}}$ was frequency-dependent. However, ${\mathrm{k}}^{\mathrm{^{\prime}}}$ subsequently decreased as hydration was further reduced to ≈ 0%. In addition, at ≈ 0% the frequency-dependency of ${\mathrm{k}}^{\mathrm{^{\prime}}}$ was less clear. This finding, therefore, implies that the ability of cartilage to store energy (available for subsequent recoil following loading), as well as the frequency-dependency of this ability to store energy for recoil, is dependent on its water content.

A reduction in ${\mathrm{k}}^{\mathrm{^{\prime}}\mathrm{^{\prime}}}$ will result in a reduction in the energy dissipated by articular cartilage. At ≈ 0% ${\mathrm{k}}^{\mathrm{^{\prime}}\mathrm{^{\prime}}}$ ranged from 55 N/mm to 46 N/mm, approximately a quarter of the values for ${\mathrm{k}}^{\mathrm{^{\prime}}\mathrm{^{\prime}}}$ at 76%. If the swelling pressure of cartilage was solely responsible for the dissipation of energy, one might expect ${\mathrm{k}}^{\mathrm{^{\prime}}\mathrm{^{\prime}}}$ to tend to zero as water content approached zero. This study has shown this is not the case, and therefore fluid interaction with collagen may be important for energy dissipation; indeed, collagen itself may have an intrinsic ability to dissipate energy. This is in agreement with Sadeghi et al. [22], who performed DMA at low frequencies (0.001 Hz) to allow time for fluid dissipative effects to occur (following time-scales of loading which mimicked those necessary to achieve peak pressure). If fluid alone was responsible for dissipation of energy, ${\mathrm{k}}^{\mathrm{^{\prime}}\mathrm{^{\prime}}}$ would be expected to increase at low frequency; however, Sadeghi et al. demonstrated this not the case, with ${\mathrm{k}}^{\mathrm{^{\prime}}\mathrm{^{\prime}}}$ being frequency independent at loading frequencies well below those which are relevant during normal gait.

The results reported by Pearson & Espino [20] for hyper- and hypo-hydration levels tested were likely in the range between 76% and 8.5%, given the test methods employed and the increase reported for ${\mathrm{k}}^{\mathrm{^{\prime}}}$. However, it is unlikely that their hyper-hydrated samples achieved 100% hydration or that their hypo-hydrated samples achieved the low levels used in this study at RH-30%. A comparison of the hyper-hydrated storage and loss values measured by Pearson & Espino and values at RH-100% in this study, show comparable results [20]. Pearson & Espino reported that they measured no statistically significant change in sample thickness; from their results, it is estimated that their samples underwent a 1 – 5% reduction in mass through dehydration, much lower than the 67% reduction in mass found in this study at RH-30%. This suggests that their values for hyper- and hypo-hydration would have been measured at hydration levels just above and below physiological conditions, respectively. When comparing this study to Pearson & Espino [20], it is important to note that they tested specimens on-bone, as opposed to the off-bone cartilage cores used in this study. The attachment to the underlying subchondral bone has been shown to alter ${\mathrm{k}}^{\mathrm{^{\prime}}\mathrm{^{\prime}}}$ and its frequency-dependency [18]*.* Restraining cartilage may also affect its ability to dissipate energy [38].

To date, there have been few studies of the relationship between hydration and the dynamic mechanical behaviour of articular cartilage. As Pearson et al. analysed two values of hydration, it is not possible to evaluate any non-linear trends in viscoelastic properties that may be present over a wider range [20]. The effect of osmolarity on the viscoelastic properties has been studied, showing an increase in dynamic modulus as osmolarity was decreased from approximately physiological (0.2 M) to 0.0015 M [39]. Cartilage water content was not reported but it is likely that this corresponds to an approximate range of water content from physiological to hyper-hydrated. Although this is similar in range to Pearson et al., the differences in methodology limit the direct comparison of data from those studies. Certainly, the technique used in this current study enables the water content of cartilage to be directly controlled, and for its effect on the viscoelastic behaviour of cartilage to be evaluated. There remains scope to evaluate the non-linearity in viscoelastic properties in between the range of hydration parameters evaluated in our current study, and the effects of hydration on the underlying subchondral bone.

It is important to note that our current study has not directly replicated the in-vivo physiological conditions of articular cartilage. For example, this study has ignored the water exchange between living cartilage and the synovial fluid which may affect surface lubrication and, therefore, the mechanical response. Surface lubrication is tangential to this current study; with the role of surface proteins being important [40, 41] and of interest to consider for future work. However, this study has carefully controlled experimental parameters in order to reduce variability between samples tested, isolating the role of water on the viscoelastic properties of cartilage.

An increase in density of cartilage substrate has been shown to increase ${\mathrm{k}}^{\mathrm{^{\prime}}}$ and ${\mathrm{k}}^{\mathrm{^{\prime}}\mathrm{^{\prime}}}$ in this study. Further, the ratio of the ability of cartilage to store/dissipate energy (${\mathrm{k}}^{\mathrm{^{\prime}}}/{\mathrm{k}}^{\mathrm{^{\prime}}\mathrm{^{\prime}}}$ ratio) on a substrate of density 156.8 kg/m^3^ was 10.0 at 1 Hz and 18.2 at 88 Hz. This increased to 12.7 at 1 Hz and 19.0 at 88 Hz when the substrate density increased to 663.7 kg/m^3^. Therefore, the potential to store excessive energy increases with the density of underlying material. This finding is in agreement with recent studies such as that by Mahmood et al. which found an increased predisposition of cartilage to fail at frequencies above those of normal gait when combined with an increased subchondral bone density [35]. Our findings are also in broad agreement with those by Fell et al., who found a positive correlation between loss modulus of cartilage and subchondral bone density [34]. However, the findings by Fell et al. may also relate to remodelling of bone and cartilage which are not the subject of this current study.

Although the densities of substrate chosen in this study do not model the properties of healthy and osteoporotic bone, they have been chosen as they enable density to be varied in a controlled manner. It has been previously shown that a variation of subchondral bone density correlates with a variation in the mechanical properties of the corresponding cartilage [34]. This results in two dependant variables, with both the mechanical behaviour of the cartilage and the underlying subchondral bone varying between samples, making it difficult to draw conclusions about the impact of only one of these variables. Therefore, in this study, we have aimed to isolate the effect of only a change in substrate density. As the substrate used in this study is a synthetic material of a known density it has not been tested independently. Using this idealised scenario, a recent study evaluated the effect of substrate density on cartilage surface damage [35].

Both an increase in BMD (through subchondral sclerosis), and an increase in water content, are believed to occur during the early onset of OA [42]. An increase in subchondral BMD might occur in response to changes in mechanotransduction [43], however, sclerosis of subchondral cortical bone may also occur without mechanical derangement [44]. From this current study, the density of a substrate under articular cartilage clearly affects the ability to dissipate energy of the cartilage-substrate structure. In the short term, increased density of the subchondral bone aiding the dissipation of energy may be advantageous, as this may reduce damage induced in cartilage, which is less capable of repairing itself compared with bone. However, if an increase in BMD is chronic, it may in the long-term increase the predisposition to failure of cartilage. If subsequent changes in cartilage include increased water content, then cartilage might be further at risk of mechanical failure. It is important to note that stress induced damage due to repetitive, over-loading is not the only potential factor when looking at the prognosis of OA, with metabolic factors implicated in the matrix-metalloproteinase related weakening of the collagen structure [45].

This current study has shown that the water content of cartilage affects the ability of cartilage both to store and to dissipate energy. It is, therefore, likely that stress transfer between collagen and its surrounding matrix [46–48] (with proteoglycans attracting water), with energy stored and dissipated during this process, dictates the viscoelastic behaviour of cartilage. However, it should be noted that other factors may cause energy dissipation in the cartilage-bone construct. For example, Becher et al. have shown that the intra-articular temperature increased by 6.1 °C after 60 min of jogging [49], whilst modelling of the knee joint suggested a potential cartilage temperature increase of 1.2 °C after 10 min of loading under conditions expected whilst walking [50]. To date, cartilage temperature changes have not been measured during dynamic loading and could provide further insight in future work. Although the water content of cartilage may increase, changing the hydration of healthy cartilage is an oversimplification of the pathogenesis of OA. For example, the increased water observed during early-onset OA is due to altered synthesis of proteoglycans [51], as opposed to saturating the proteoglycans in healthy cartilage. Better understanding of the mechanisms by which water is physically held within cartilage, and their alteration during OA, may benefit further understanding of the mechanical behaviour of both healthy and OA cartilage. Although this study has not aimed to mimic the physiological conditions of cartilage, it has provided further insight into the effects of these variables on the mechanical behaviour of cartilage. The exploration and understanding of the relationships between the viscoelastic properties of cartilage, and parameters such as hydration and substrate density could aid in understanding the mechanical behaviour of osteoarthritic cartilage in a controlled manner [52] and allow a more targeted approach to cartilage repair or the design of bioinspired materials [53].

### Conclusion

This study has found that a decrease in hydration will cause a decrease in the loss stiffness of articular cartilage and that a non-linear relationship may exist between hydration and the storage stiffness of cartilage. This study also found that both storage and loss stiffnesses of cartilage increase as the substrate density increases, which suggests greater likelihood of cartilage failure with increasing density of the underlying bone.

## Supplementary Information

**Additional file 1.** SupplementaryData.

## Data Availability

The datasets used and/or analysed during the current study are available from the corresponding author on reasonable request.
