# Supplementary material for: Analysis of hydration and subchondral bone density on the viscoelastic properties of bovine articular cartilage
Source: BMC Musculoskelet Disord. 2022 Mar 8;23:228. doi: 10.1186/s12891-022-05169-0 (PMC8905800; doi:10.1186/s12891-022-05169-0)
Supplement: Supplementary file 1 — Additional file 1. SupplementaryData. [file 12891_2022_5169_MOESM1_ESM.docx]

**SUPPLEMENTARY DATA**

**S.1 Substrate Control data**


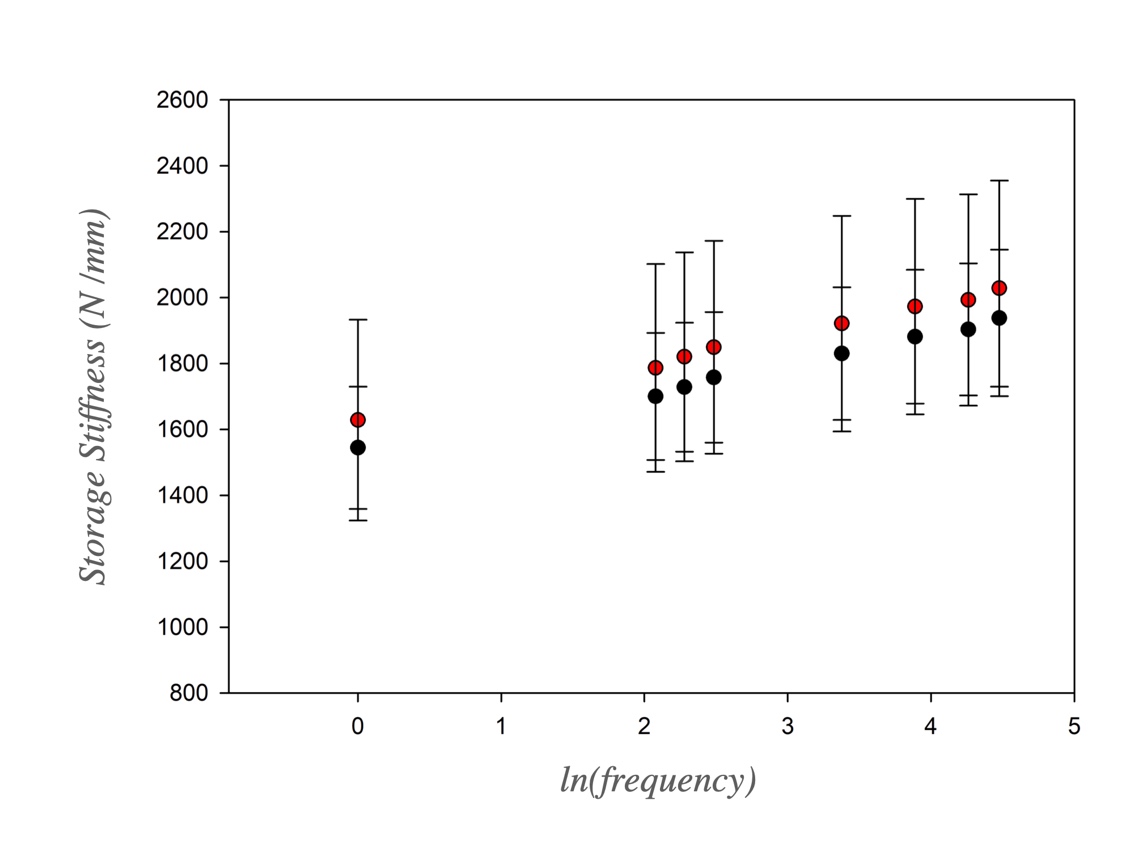


**Figure S1a**: Mean Storage stiffness as a function of the Natural log(frequency; f) of cartilage samples (black), and then repeat tested (red) after 4 hours. Mean values have been calculated from N = 8 samples Error bars show 95% confidence intervals.


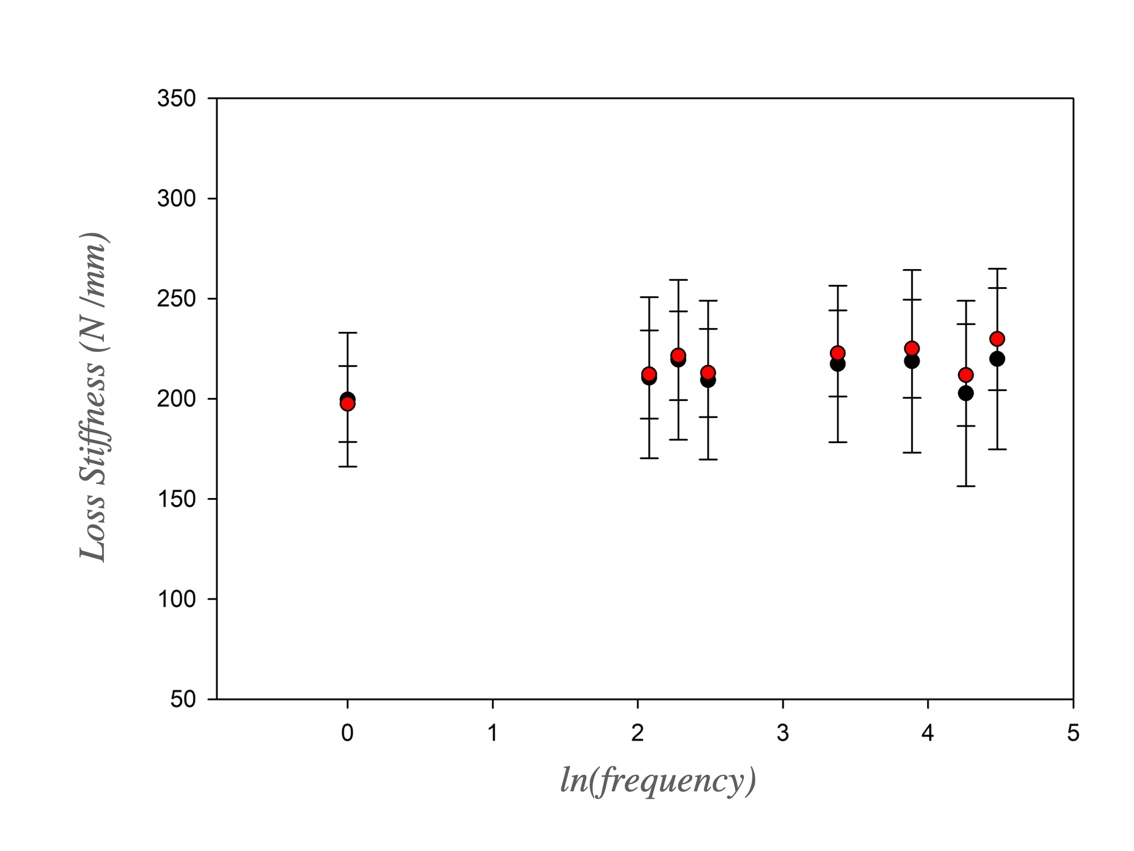


**Figure S1b**: Mean Loss stiffness as a function of the Natural log(frequency; f) of cartilage samples (black), and then repeat tested (red) after 4 hours. Mean values have been calculated from N = 8 samples Error bars show 95% confidence intervals.

**S.2 Hydration Control data**

Control data is provided for loss (Figure S.2) and storage stiffness (Figure S.3), along with a plot of the relevant results corrected as per control data (Figure S.4). The overall trends predicted from the original data and following correction using control data, have not altered (Figure S.5).


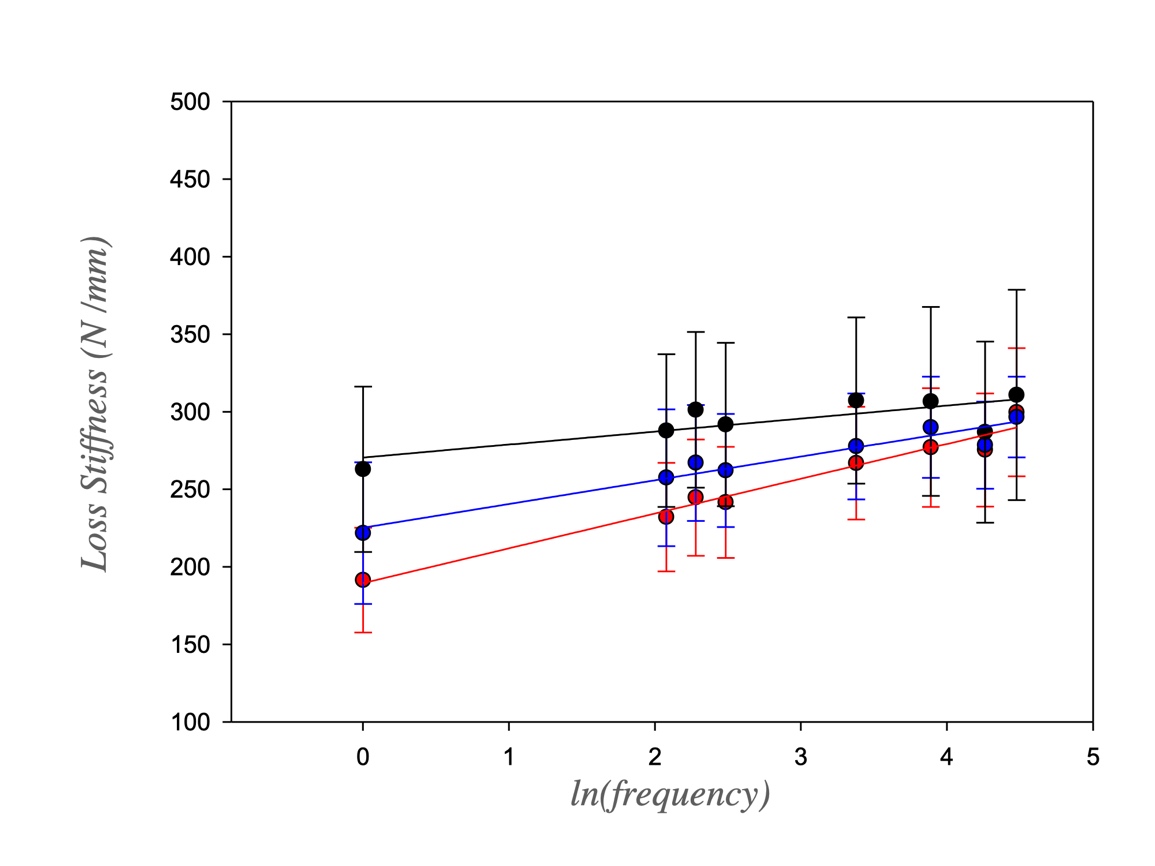


Figure S.2. Loss Stiffness for control samples (n=8) tested after 24 hours (red), 48 hours (blue), and 72 hours (black). Samples were stored in a hydration chamber at RH-100% between tests.


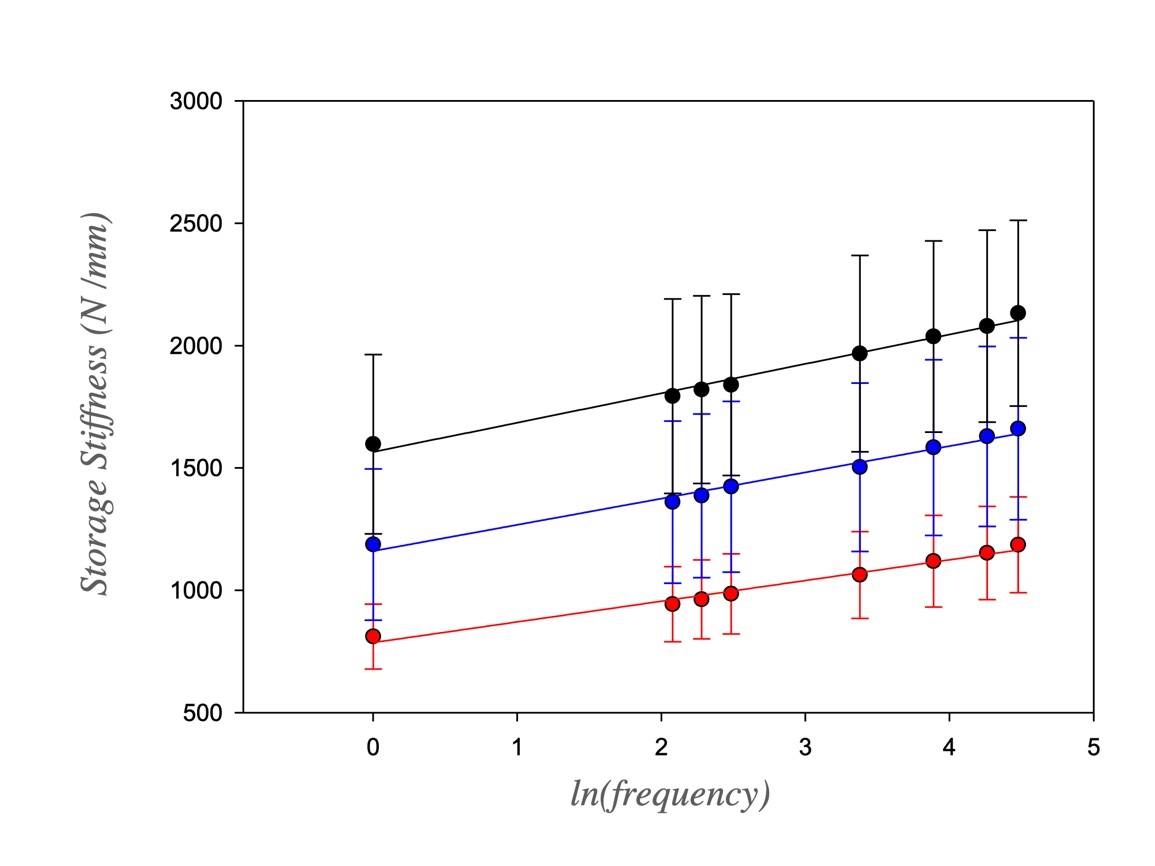


Figure S.3: Storage Stiffness for control samples (n=8) tested after 24 hours (red), 48 hours (blue), and 72 hours (black). Samples were stored in a hydration chamber at RH-100% between tests.


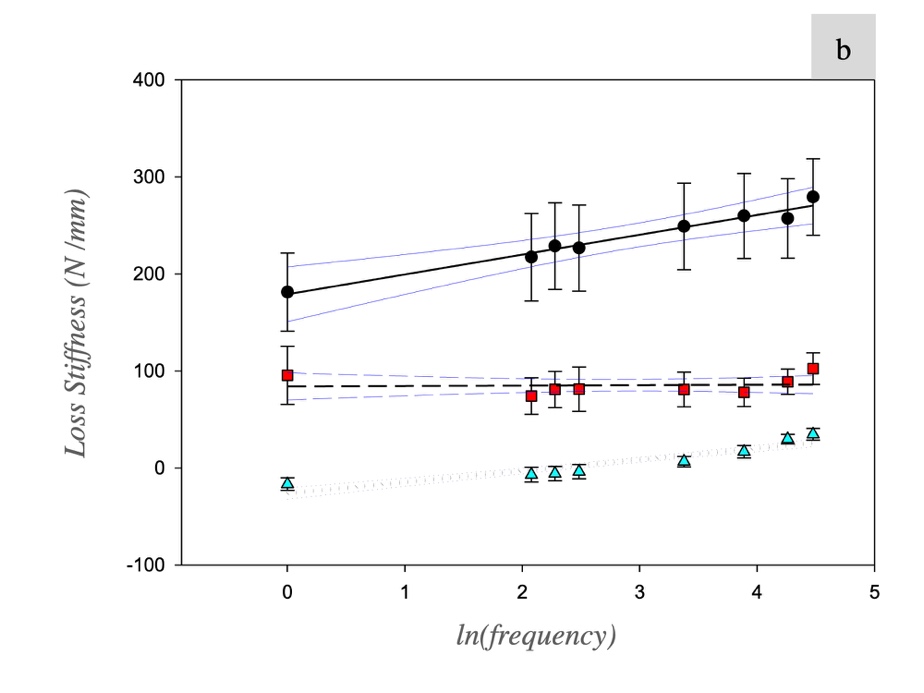

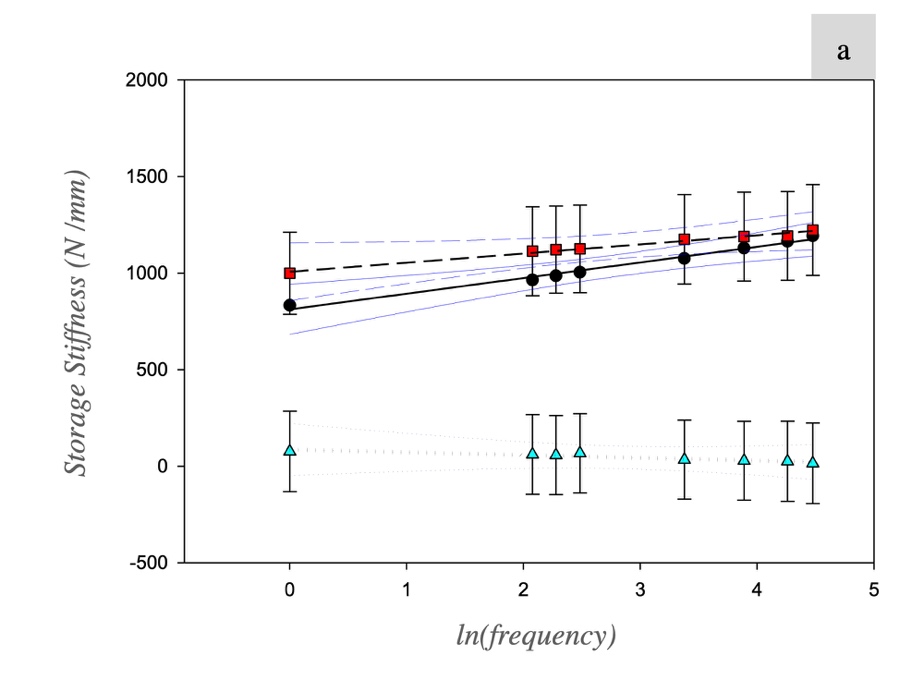


**Figure S.4**: Corrected mean stiffness as a function of the Natural log(frequency) for **(a)** Storage stiffness and **(b)** loss stiffness of cartilage samples at three different hydration levels; RH-100% (black), RH-30% (red), and RH-1% (cyan). Mean values have been calculated from n = 16 samples. Error bars show 95% confidence intervals for N = 8 independent samples, blue lines show 95% confidence intervals for the lines of regression for N = 8 independent samples.

**Figure S.5:** Corrected (black) and uncorrected (grey) storage stiffness against cartilage water content for **(a)** 1 Hz and **(b)** 90 Hz. Corrected data for 0% water content at 1 Hz has been capped to a minimum storage stiffness value of 0 N/mm.


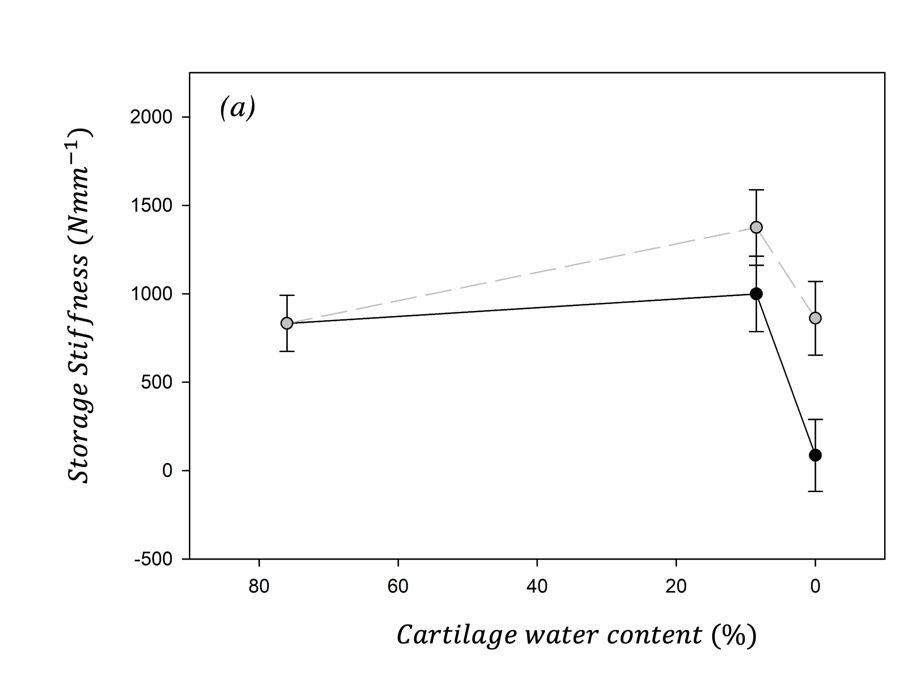

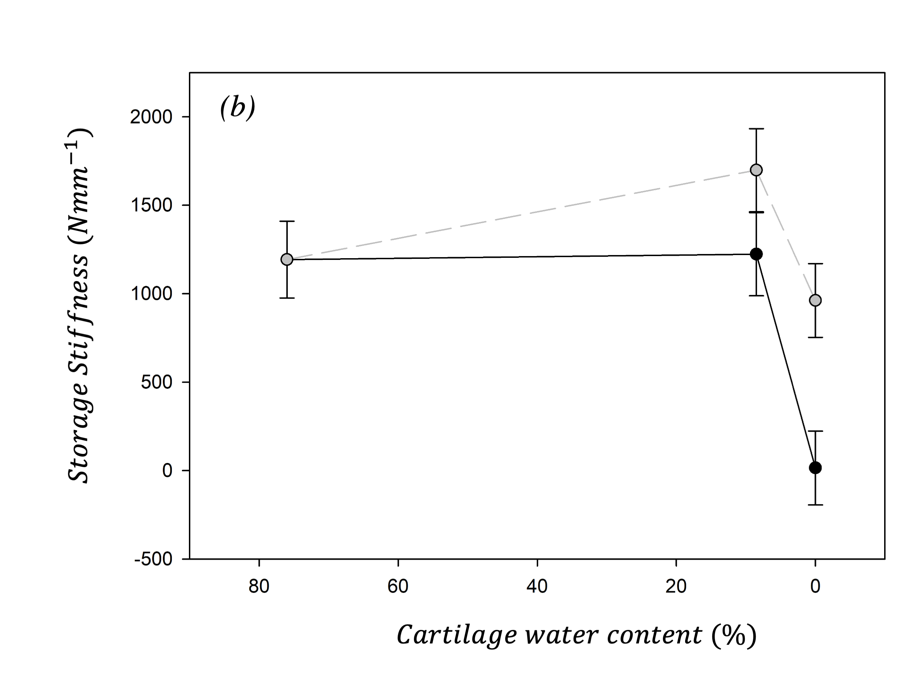


**Figure S.6**: Corrected (black) and uncorrected (grey) loss stiffness against cartilage water content for **(a)** 1 Hz and **(b)** 90 Hz. Corrected data for 0% water content at 1 Hz has been capped to a minimum loss stiffness value of 0 N/mm.


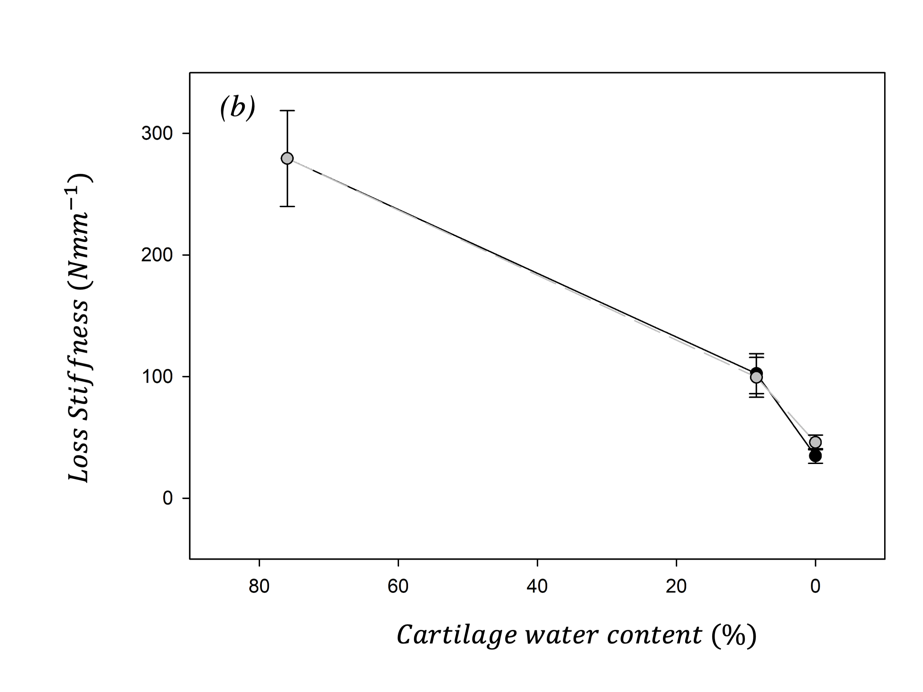

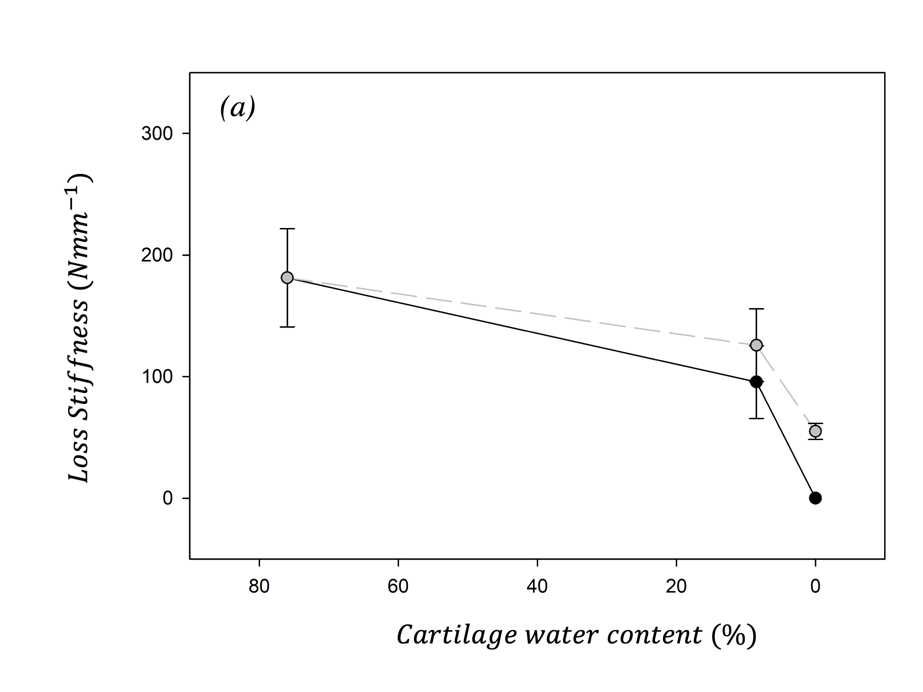


**S.3 Test data.**

Data sets for storage and loss stiffness, for tests relating to different substrates, as a mean per frequency are provided (Table S.1) along with data for each sample which describe their frequency dependent trends (Table S.2). The equivalent data is also provided for hydration (Table S.3 and S.4).

**Table S.1.** Storage and Loss stiffness of cartilage tested on two densities of synthetic bone at different loading frequencies. Mean and Standard Deviation (SD) calculated for 24 tissue samples.

| **Freq (Hz)** | $\boldsymbol{k}^{\boldsymbol{'}}$ **(N/mm)** | | | | $\boldsymbol{k}^{\boldsymbol{''}}$ **(N/mm)** | | | |
| --- | --- | --- | --- | --- | --- | --- | --- | --- |
|  | **High Density** | | **Low Density** | | **High Density** | | **Low Density** | |
|  | **Mean** | **SD** | **Mean** | **SD** | **Mean** | **SD** | **Mean** | **SD** |
| **1** | 1180 | 120 | 380 | 46 | 92.8 | 13 | 38.3 | 6.0 |
| **8** | 1280 | 130 | 421 | 51 | 81.5 | 14 | 30.9 | 4.2 |
| **10** | 1300 | 130 | 427 | 51 | 85.0 | 14 | 31.0 | 4.0 |
| **12** | 1310 | 130 | 431 | 52 | 78.1 | 14 | 29.3 | 3.8 |
| **29** | 1350 | 130 | 444 | 53 | 77.3 | 13 | 28.1 | 3.6 |
| **49** | 1370 | 130 | 453 | 54 | 74.0 | 13 | 26.8 | 3.4 |
| **71** | 1390 | 130 | 459 | 55 | 66.6 | 14 | 24.0 | 3.1 |
| **88** | 1400 | 130 | 463 | 55 | 73.6 | 14 | 25.5 | 3.07 |

**Table S.2.** Storage stiffness constants (A and B) for Equation. 5, and loss stiffness constants (C and D) for Equation. 6, for all 24 samples used in substrate density experiment.

| **Sample Number** | $\boldsymbol{k}^{\boldsymbol{'}}$ | | | | $\boldsymbol{k}^{\boldsymbol{''}}$ | | | |
| --- | --- | --- | --- | --- | --- | --- | --- | --- |
|  | **A (N/mm)** | | **B (N/mm)** | | **C (N/mm)** | | **D (N/mm)** | |
|  | **High density** | **Low Density** | **High Density** | **Low Density** | **High density** | **Low Density** | **High Density** | **Low Density** |
| 1 | 41.3 | 19.1 | 1210 | 438 | -7.11 | -3.64 | 82.9 | 40.2 |
| 2 | 49.1 | 20.9 | 1260 | 470 | -3.56 | -3.91 | 93.5 | 43.2 |
| 3 | 49.1 | 20.9 | 1240 | 381 | -8.29 | -4.1 | 117 | 48.3 |
| 4 | 44.1 | 20.9 | 1150 | 406 | -3.87 | -3.43 | 84.5 | 43.2 |
| 5 | 44.4 | 18.7 | 1220 | 405 | -3.61 | -2.81 | 78.4 | 37.1 |
| 6 | 43.9 | 19.8 | 1100 | 400 | -2.29 | -2.85 | 81.2 | 38.9 |
| 7 | 37.1 | 20 | 959 | 424 | -1.64 | -3.87 | 61.8 | 43.1 |
| 8 | 35.2 | 19.7 | 1220 | 456 | -4.58 | -3.59 | 67.5 | 40.3 |
| 9 | 48.6 | 20.9 | 1280 | 456 | -8.91 | -3.55 | 106 | 42.8 |
| 10 | 49.4 | 17.5 | 862 | 326 | 1.26 | -1.58 | 99 | 36.2 |
| 11 | 53.6 | 15.5 | 1130 | 337 | -2.12 | -1.83 | 101 | 30.4 |
| 12 | 50 | 15.2 | 1160 | 333 | -3.35 | -2.02 | 98 | 30.4 |
| 13 | 55.2 | 15.8 | 1140 | 345 | -5.12 | -2.31 | 112 | 32.4 |
| 14 | 50.5 | 15.8 | 1050 | 317 | -3.63 | -1.92 | 96.2 | 32.8 |
| 15 | 50.6 | 14.6 | 1090 | 322 | -5.41 | -2.08 | 95.1 | 30 |
| 16 | 49.3 | 17.7 | 1230 | 360 | -6.07 | -3.25 | 92.3 | 38.2 |
| 17 | 47 | 16.9 | 1330 | 372 | -6.58 | -2.63 | 87.9 | 34 |
| 18 | 47.5 | 15.5 | 1280 | 350 | -6.69 | -2.43 | 83.8 | 31 |
| 19 | 50 | 18.4 | 1270 | 366 | -5.4 | -3.43 | 94.3 | 40.3 |
| 20 | 51.1 | 22.9 | 1260 | 421 | -9.65 | -4.27 | 116 | 49.4 |
| 21 | 53.5 | 19.5 | 1270 | 403 | -7.11 | -3.14 | 105 | 40.1 |
| 22 | 47.2 | 18.7 | 1440 | 413 | -5.27 | -3.03 | 84.1 | 37.6 |
| 23 | 51.1 | 14.9 | 1180 | 351 | -3.52 | -2.06 | 92.2 | 29.3 |
| 24 | 51.9 | 15.8 | 1260 | 339 | -7.64 | -2.91 | 106 | 34.2 |
| **Mean** | **47.8** | **18.1** | **1190** | **383** | **-5.01** | **-2.94** | **93.1** | **37.6** |
| **SD** | **4.79** | **2.32** | **119** | **45.3** | **2.49** | **0.766** | **13.5** | **5.61** |

**Table S.3.** Storage and Loss stiffness properties of cartilage after equilibration at a relative humidity of 100%, 30%, and 1%, at different loading frequencies. Mean and Standard Deviation (SD) calculated for 16 tissue samples.

| **Freq (Hz)** | $\boldsymbol{k}^{\boldsymbol{'}}$ **(N/mm)** | | | | | | $\boldsymbol{k}^{\boldsymbol{''}}$ **(N/mm)** | | | | | |
| --- | --- | --- | --- | --- | --- | --- | --- | --- | --- | --- | --- | --- |
|  | **RH 100%** | | **RH 30%** | | **RH 1%** | | **RH 100%** | | **RH 30%** | | **RH 1%** | |
|  | **Mean** | **SD** | **Mean** | **SD** | **Mean** | **SD** | **Mean** | **SD** | **Mean** | **SD** | **Mean** | **SD** |
| **1** | 833 | 220 | 1380 | 350 | 862 | 380 | 181 | 56 | 125.8 | 49 | 55.0 | 12 |
| **8** | 965 | 250 | 1530 | 370 | 911 | 380 | 217 | 63 | 99.5 | 31 | 49.0 | 11 |
| **10** | 986 | 260 | 1550 | 360 | 914 | 380 | 229 | 63 | 103 | 31 | 50.8 | 11 |
| **12** | 1010 | 260 | 1560 | 370 | 921 | 380 | 227 | 63 | 102 | 38 | 46.4 | 11 |
| **29** | 1080 | 280 | 1620 | 370 | 938 | 380 | 249 | 66 | 91.6 | 31 | 47.0 | 9.9 |
| **49** | 1130 | 280 | 1650 | 370 | 947 | 380 | 260 | 65 | 90.9 | 22 | 46.5 | 10.8 |
| **71** | 1160 | 300 | 1670 | 370 | 952 | 390 | 257 | 64 | 91.9 | 27 | 41.2 | 9.4 |
| **88** | 1190 | 300 | 1700 | 370 | 961 | 390 | 279 | 62 | 99.4 | 25 | 45.9 | 9.9 |

| **Sample Number** | $\boldsymbol{k}^{\boldsymbol{'}}$ | | | | | | $\boldsymbol{k}^{\boldsymbol{''}}$ | | | | | | |
| --- | --- | --- | --- | --- | --- | --- | --- | --- | --- | --- | --- | --- | --- |
|  | **A (N/mm)** | | | **B (N/mm)** | | | **C (N/mm)** | | | **D (N/mm)** | | | |
|  | RH 100% | RH 30% | RH 1% | RH 100% | RH 30% | RH 1% | RH 100% | RH 30% | RH 1% | RH 100% | RH 30% | RH 1% |  |
| 1 | 69.8 | 55 | 20.7 | 881 | 1110 | 582 | 18.5 | -4.72 | -3.25 | 132 | 85.8 | 77.1 |  |
| 2 | 56.4 | 56.7 | 27.4 | 630 | 1870 | 1750 | 23.2 | -9.4 | -3.83 | 134 | 131 | 57.4 |  |
| 3 | 86.4 | 80.7 | 41 | 859 | 1210 | 575 | 23.5 | -3.18 | -1.28 | 177 | 96.9 | 63.3 |  |
| 4 | 62.2 | 70 | 19.8 | 730 | 1180 | 833 | 18.6 | -0.848 | -1.61 | 142 | 84.9 | 52.9 |  |
| 5 | 88 | 44.4 | 19.1 | 832 | 1030 | 492 | 31.9 | 7.57 | -1.96 | 197 | 59 | 56.7 |  |
| 6 | 96.1 | 54.5 | 19.5 | 1160 | 771 | 950 | 10.5 | -1.01 | -2.89 | 167 | 71.5 | 60.1 |  |
| 7 | 51.2 | 113 | 29.5 | 461 | 1430 | 873 | 21.7 | -19.5 | -4.01 | 110 | 219 | 55 |  |
| 8 | 124 | 62.5 | 16.5 | 716 | 1380 | 688 | 33 | -2.43 | -1.22 | 246 | 97.6 | 51.7 |  |
| 9 | 121 | 69.7 | 15.5 | 1220 | 1170 | 450 | 11.5 | -6.83 | -3.39 | 312 | 114 | 47.7 |  |
| 10 | 103 | 52 | 15.2 | 957 | 1590 | 702 | 20.4 | -2.27 | -2.38 | 265 | 80.6 | 47.1 |  |
| 11 | 45.5 | 71.3 | 18.2 | 689 | 1260 | 1280 | 14.6 | -9.37 | -3.84 | 109 | 139 | 62.7 |  |
| 12 | 54.2 | 68.6 | 11.1 | 530 | 2220 | 710 | 17.1 | -23.2 | -0.878 | 124 | 206 | 34.5 |  |
| 13 | 88.5 | 86.3 | 33.5 | 1090 | 1770 | 884 | 17.4 | -16.8 | -4.31 | 203 | 185 | 75.1 |  |
| 14 | 91.3 | 47.5 | 15.9 | 903 | 1490 | 542 | 19.5 | -2.99 | -3 | 211 | 79.7 | 53.6 |  |
| 15 | 72.1 | 105 | 23.2 | 700 | 1180 | 943 | 21 | -9.97 | -0.733 | 157 | 156 | 44.8 |  |
| 16 | 85.5 | 81.1 | 16.3 | 637 | 1440 | 1590 | 24.4 | -2.7 | -0.176 | 180 | 109 | 34.2 |  |
| **Mean** | **81** | **69.9** | **21.4** | **813** | **1380** | **865** | **20.4** | **-6.72** | **-2.42** | **179** | **120** | **54.6** |  |
| SD | **23** | **19** | **7.6** | **211** | **344** | **367** | **6** | **7.6** | **1.3** | **56.1** | **47.6** | **11.6** |  |

**Table S.4.** Storage stiffness constants (A and B) for Equation. 5, and loss stiffness constants (C and D) for Equation. 6, for all 16 samples used in hydration experiment.
